# Understanding Adaptation in Large Populations

**DOI:** 10.1371/journal.pgen.1000987

**Published:** 2010-06-17

**Authors:** Nick Barton

**Affiliations:** Institute of Science and Technology (IST) Austria, Klosterneuburg, Austria; Fred Hutchinson Cancer Research Center, United States of America

For the past half-century, population genetics has been dominated by studies of molecular evolution, interpreted under the neutral theory. This predicts that the rate of substitution equals the rate of neutral mutation and that the genetic diversity within populations depends on the product of population size and neutral mutation rate, 4Nμ. Yet, diversity clearly does not increase in direct proportion to population size [1], [2]. Bacterial populations are typically more diverse than insects, which in turn are more diverse than mammals, but these differences span only an order of magnitude, even though actual population sizes vary far more. We can see that the standard neutral theory makes no sense for very abundant species; it predicts that genes share common ancestry 2N generations back, which may often be older than the species, and, for microbes, older than the planet itself.

In abundant species, conventional random drift must be negligible; diversity is instead limited by occasional drastic bottlenecks and by recurrent selective sweeps [3]. Often, the net rate of such sporadic events is described by defining an “effective size,” N_e_, which is much smaller than the actual census size. However, this effective size is only a description of the level of neutral diversity and does not tell us how random drift influences the adaptive alleles that actually matter to the organism. It is crucial to distinguish, here, between short-term factors such as sex ratio or variance in offspring number that increase the rate of random drift, and more drastic events such as bottlenecks or selective sweeps that affect the whole population. The former may reduce the short-term effective population size by as much as an order of magnitude below the census number [4], but nevertheless, random drift will be negligible if the census number is sufficiently high. In contrast, selective sweeps and severe bottlenecks are essentially independent of the typical population number and limit neutral diversity in the long term.

Karasov et al. [5] use insecticide resistance in *Drosophila melanogaster* to give a detailed and elegant example of how the pattern of adaptation depends on population size in the short term, and is independent of whatever long-term factors determine neutral diversity. Resistance to organophosphate insecticides is due to specific amino-acid changes in the active site of the target enzyme acetyl-cholinesterase, with the most resistant alleles having three changes. Karasov et al. show that although the same amino acids (indeed, because of constraints from the genetic code, the same nucleotide changes) are always involved, these have arisen independently many times on different local haplotypes. Most striking is that complex resistance alleles have arisen through successive mutations, with no need for recombination, and all within 50 years, or ∼1000 generations.

Such rapid and repeated change is inexplicable if the population size is around 10^6^, the effective number inferred from neutral diversity. Then, assuming a rate of mutation to a specific nucleotide of ∼10^−8^/3 (there are three possibilities at each site), the appropriate mutation would arise only every ∼150 generations, and most such mutations would be lost by chance. In fact, there may be more than 10^6^ *D. melanogaster* in a single orchard, so that every possible nucleotide change arises in every generation, within any local area. Most such mutations will be lost—their chance of establishment is roughly twice their selective advantage, 2S—but nevertheless, multiple favourable mutations will start to increase, carrying with them unique blocks of genome. These various mutations may differ slightly in fitness because they will be associated with different deleterious alleles. However, because only a short segment of genome will hitch-hike with the favoured allele, this linked load will be small. Eventually, the favoured allele will fix everywhere and will be associated with a surrounding genome whose diversity depends only on the number of favourable mutations that enter in every generation, 2Nμ, and whose length depends on the inverse of the time to fixation, ∼S/log(S/μ) (Figure 1).

**Figure 1:**
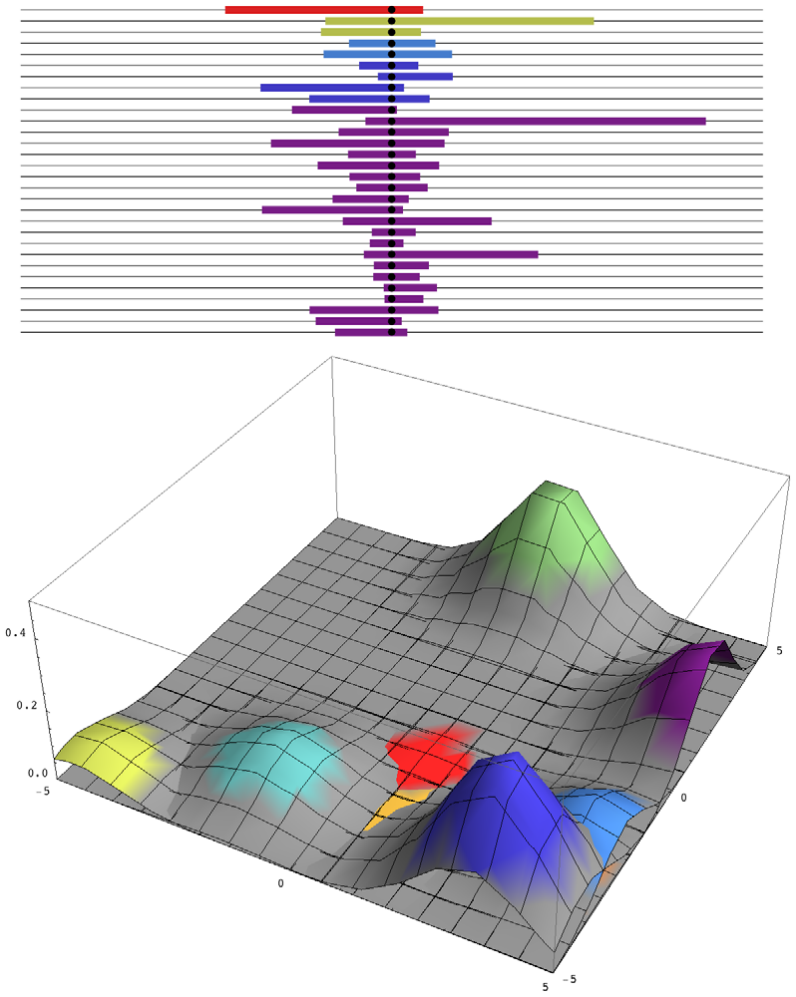
Patterns of diversity across the genome and in space. Top: A sample of 30 genomes, taken immediately after completion of a selective sweep, contains five distinct haplotypes. The horizontal axis represents the genome, with the selected site at the centre, and the unique block of haplotype carried by each independent mutation is indicated by the coloured segment. Over time, these segments will recombine with eachother, so that the region of strong linkage disequilibrium will shrink, without altering the region of reduced diversity. Bottom: A snapshot of the two-dimensional spatial distribution during the process of fixation. The nine commonest alleles are shown, which together make up 93% of the total diversity in the population. Colours correspond to those above. How many independent mutations contribute to adaptation depends only on the number of favourable mutations that enter in every generation, 2Nμ. In this example, 2Nμ = 2; simulations with smaller 2Nμ show contributions from correspondingly fewer mutations.

In very large populations, the distinction between adaptation from new mutations—invoked here by Karasov et al. [5]—and adaptation from standing variation becomes blurred. If there is selection s against resistance alleles before insecticide is applied, then resistance alleles will be present at a frequency of ∼µ/s, and each allele will have originated on average ∼1/s generations back; in this example, Karasov et al. estimate 1/s to be ∼5–20 generations. Once insecticide is present, giving resistance alleles an advantage S, any such allele has a chance of being picked up if it occurs within ∼1/S generations; those that occur much later will remain rare, relative to the first mutations. So, standing variation and new mutations are both expected to contribute in the ratio of 1/*s*∶1/*S*. Pennings and Hermisson [6]–[8] give a detailed analysis of this issue. However, whether the resistance alleles were present before they became advantageous or arose soon afterwards makes no tangible difference: the key point is that, in a very large population, multiple mutations are likely to contribute to adaptations, which changes the signal that they leave in the surrounding DNA sequence.

In other examples, we can see the diverse origins of adaptive alleles in their geographic pattern. For example, in the late 1950s, there were several outbreaks of resistance to the anticoagulant poison warfarin in British rats, each corresponding to the establishment of a different resistance allele at the target locus [9]. In humans, the best examples of molecular adaptation are from the several mechanisms of malaria resistance, which show an overlapping mosaic of alleles at different genes; within β-globin, different amino-acid changes confer resistance in different places, and the best-known sickle-cell allele itself has multiple origins [10]. The example of organophosphate resistance described by Karasov et al. is especially clear because of the short timescale, because precisely the same nucleotide substitutions have increased many times, and because of the detailed analysis of the surrounding haplotype structure.

The complexity of molecular adaptation should change our view of the “molecular clock”—one of the two pillars of the neutral theory. The excess of divergence over polymorphism suggests that, in many organisms, a large fraction of amino-acid substitutions are due to positive selection; at least as many non-coding differences may also be driven by selection [11]. If such adaptive substitutions follow a change in environment in a very large population, then each will involve many mutations, rather than just one. In addition, a change in environment may trigger multiple substitutions, both because several changes are individually favoured, as here, and because one substitution may make others become favourable (i.e., epistasis; [12], [13]). This helps to explain why rates of molecular evolution vary, implying that substitutions are strongly clustered [14]. The complexity of this process makes it hard to understand why the rate of the molecular clock is even roughly equal to the mutation rate, as observed and as is expected from the most naive version of the neutral theory.

If populations were really as small as is implied by the effective sizes inferred from neutral diversity, then they would adapt much less effectively: *Drosophila* would take far longer to evolve resistance to insecticides, for example. In large populations, weakly selected alleles are still vulnerable to sporadic bottlenecks and selective sweeps, but strongly favoured mutations are hardly affected, and so can be picked up by selection even if neutral diversity is low. On this view, the ability of populations to adapt under strong selection depends on the actual number of favourable mutations that arise in each generation, which cannot be estimated by studying neutral markers. To understand adaptation, we need more studies such as this, which focus on adaptation itself.

## References

[1] Lewontin RC (1974). The genetic basis of evolutionary change.

[2] Nevo E (1988). Genetic diversity in nature - patterns and theory.. Evol Biol.

[3] Maynard Smith J, Haigh J (1974). The hitch-hiking effect of a favourable gene.. Genet Res.

[4] Frankham R (1995). Effective population size/adult population size ratios in wildlife: a review.. Genet Res.

[5] Karasov T, Messer SW, Petrov DA (2010). Evidence that adaptation in *Drosophila* is not limited by mutation at single sites.. PLoS Genet.

[6] Hermisson J, Pennings PS (2005). Soft sweeps: molecular population genetics of adaptation from standing genetic variation.. Genetics.

[7] Pennings PS, Hermisson J (2006). Soft sweeps II - Molecular population genetics of adaptation from recurrent mutation or migration.. Mol Biol Evol.

[8] Pennings PS, Hermisson J (2006). Soft sweeps III: the signature of positive selection from recurrent mutation.. PLoS Genet.

[9] Drummond D (1966). Rats resistant to warfarin.. New Sci.

[10] Oner C, Dimovski AJ, Olivieri NF, Schiliro G, Codrington JF (1992). b*S* haplotypes in various world populations.. Hum Genet.

[11] Eyre-Walker A, Keightley PD (2009). Estimating the rate of adaptive molecular evolution in the presence of slightly deleterious mutations and population size change.. Mol Biol Evol.

[12] Gillespie JH (1984). Molecular evolution over the mutational landscape.. Evolution.

[13] Orr HA (2005). The genetic theory of adaptation: a brief history.. Nat Rev Genet.

[14] Gillespie JH (1991). The causes of molecular evolution.

